# Characterizing the relationships between dietary indices, gallstone prevalence and the need for gallbladder surgery in the general US population

**DOI:** 10.3389/fnut.2024.1392960

**Published:** 2024-05-07

**Authors:** Chenyu Jiang, Yaojian Shao

**Affiliations:** ^1^Department of Geriatric, Taizhou Central Hospital (Taizhou University Hospital), Taizhou, Zhejiang, China; ^2^Department of Gastroenterology, Taizhou Central Hospital (Taizhou University Hospital), Taizhou, Zhejiang, China

**Keywords:** DII, CDAI, gallstone prevalence, age at gallbladder surgery, NHANES

## Abstract

**Background:**

The dietary inflammatory index (DII) and composite dietary antioxidant index (CDAI) were developed as tools for use when seeking to assess the potential inflammatory and antioxidant activity of a given diet, respectively. The associations between these indices and gallstone incidence remain largely unexplored.

**Objective:**

The present study sought to clarify how both the DII and the CDAI are related to gallstone incidence and age at first gallbladder surgery among adults in the USA.

**Methods:**

Cross-sectional data from the 2017–2020 cycles of the National Health and Nutrition Examination Survey (NHANES) pertaining to 12,426 individuals were used to conduct the present study. Data from 2 days with 24-h dietary recall were employed when calculating DII and CDAI scores. Relationships between dietary indices and the incidence of gallstones were assessed through logistic regression analyses, while linear regression analyses were employed to characterize how these indices are associated with the age at first gallbladder surgery.

**Results:**

Higher DII scores and lower CDAI scores, which, respectively, denote diets with greater inflammatory potential and reduced antioxidant potential, were found to be associated with higher gallstone incidence even following adjustment for potential confounding factors. Smooth curve fitting suggested that the association between DII and gallstones was nearly linear, whereas that between CDAI and gallstone incidence was nonlinear. Higher DII values were also related to first gallbladder surgery at an earlier age (*β* = −0.64, 95% CI: −1.26, −0.02).

**Conclusion:**

These results emphasize the benefits of anti-inflammatory diets rich in antioxidants, which may help reduce gallstone incidence among adults in the USA. Higher DII scores may also predict the need for gallbladder surgery at a younger age.

## Introduction

Gallstones are lithic digestive fluid deposits that can develop in the bile ducts or gallbladder as a consequence of the presence of bile containing atypically high bilirubin or cholesterol levels (1). Also referred to as cholelithiasis, gallstones are among the most prevalent diseases of the digestive system, in addition to being the most expensive with respect to their socioeconomic impact as they have a severe negative impact on patient quality of life while requiring the consumption of extensive healthcare resources (2). Gallstones impact an estimated 10–20% of adults in the world, and while they are asymptomatic in most cases, 20% of affected patients develop gallstone disease that is characterized by cholecystitis, abdominal pain, pancreatitis, and cholangitis, often necessitating surgical removal of the gallbladder for effective management (3, 4). Gallstones can also cause localized and systemic inflammation (5) while disrupting normal metabolic activity (6, 7).

In prior studies, a wide range of gallstone-related risk factors have been identified including female sex, advanced age, dietary factors, and metabolic syndrome, characterized by a combination of insulin resistance, diabetes mellitus, obesity, and a lack of physical activity (8, 9). Diet plays a particularly important role in the incidence of gallstones, with the consumption of high levels of carbohydrates, a highly caloric diet, a high glycemic load, and low levels of fiber, vegetable, and fruit consumption all being related to this outcome (10–12). Given a growing focus on the potential importance of diet-related inflammation, the dietary inflammatory index (DII) was designed as a tool capable of capturing and representing the impact of different dietary factors on the production of inflammatory biomarkers including C-reactive protein (CRP), interleukin (IL)-1β, IL-4, IL-6, IL-10, and tumor necrosis factor (TNF)-a (13). The DII provides a comprehensive estimate of the inflammatory potential of a given diet based on the consumption of foods with anti- or pro-inflammatory effects, with higher values being indicative of a diet that is potentially more inflammatory (14, 15). The composite dietary antioxidant index (CDAI) is another index that captures information regarding the antioxidant properties of a given diet (16), with diets rich in selenium, zinc, and other antioxidants being better suited to mitigating oxidative stress, potentially leading to a reduction in gallstone incidence (17, 18).

While each of these indices has previously been leveraged to test the relationships between the inflammatory potential of an individual’s diet and their risk of gallstones (19, 20), these prior studies have been subject to limitations and there have not been any simultaneous investigations of these indices in the general US adult population. Epidemiological data are thus lacking regarding the degree to which DII and CDAI scores represent potential gallstone-related risk factors. Dietary potential is a metric that is increasingly being evaluated when conducting epidemiologic and interventional studies aimed at the treatment and management of gallstones, underscoring a need to validate the associations between these biomarkers and gallstone disease activity. This study was thus developed to separately explore how dietary profiles, as measured with DII and CDAI values, are related to gallstone prevalence and other metrics among adults in the USA.

## Method

### Study subject data

**Figure 1:**
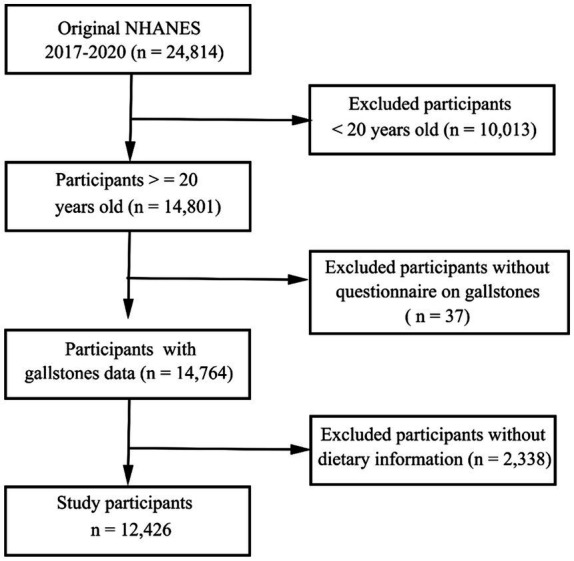
Study participant selection flow chart.

The NHANES study employs a complex methodological approach to select representative subjects from the general US population every other year. The primary goal of this ongoing study is to gauge the nutritional and health status of US participants. The National Center for Health Statistics Institutional Review Board approved the NHANES study, with all participants having provided written informed consent to participate. In total, 24,814 individuals partook in the 2017–2020 NHANES cycle. The dietary information was obtained through two 24-h dietary recall interviews to calculate the DII and CDAI score. Following the exclusion of participants for whom dietary recall or gallstone questionnaire data were missing, all remaining subjects were included in the present analysis (Figure 1).

### DII calculations

DII values provide a comprehensive system used to score the potential inflammatory properties of a given individual’s diet (13). In total, 28 different parameters were taken into consideration when calculating DII values, including energy, carbohydrates, protein, alcohol, fiber, cholesterol, total fat, saturated fat, polyunsaturated fatty acid, monounsaturated fatty acid, n-3 fatty acids, n-6 fatty acids, niacin, levels of vitamins A, B1, B2, B6, B12, C, D, and E, beta carotene, iron, magnesium, zinc, selenium, folic acid, and caffeine. Negative and positive scores were, respectively, employed for anti- and pro-inflammatory compounds. Individual scores were then summed together to yield a comprehensive DII score capturing overall dietary inflammatory potential. DII values were then used to group subjects into four quartiles.

### CDAI calculations

The CDAI serves as a composite index first developed by Wright et al. that seeks to capture information regarding dietary antioxidant intake (21). CDAI values were computed based on 24-h recall data pertaining to the intake of dietary carotenoids, selenium, zinc, and vitamins A, C, and E, normalizing the intake of these antioxidants with a zero-mean method [(daily intake of antioxidant – mean intake of antioxidant)/standard deviation] (22). Levels of standardized dietary antioxidant intake were summed together, thereby enabling CDAI calculations. CDAI scores were further used to group study participants into four quartiles.

### Other analyzed covariates

Other covariates analyzed for study participants included age (years), sex (male, female), ethnicity (Mexican American, Other Hispanic, Non-Hispanic White, Non-Hispanic Black, other), family poverty to income ratio (PIR), educational status (< high school, high school, > high school), body mass index (BMI), physical activity (no, moderate, vigorous, both [including moderate and vigorous]), energy intake (kcal), smoking status (yes/no), incidence of hypertension, hyperlipidemia, and diabetes mellitus (all yes/no) and use of contraceptives (yes/no), estrogens (yes/no), and fibrates (yes/no). BMI values were determined based on weight (kg) divided by the square of height (m^2^), and these values were used to classify individuals as being normal (<25 kg/m^2^), overweight (25 to <30 kg/m^2^), or obese (≥30 kg/m^2^). Laboratory parameters that were measured included levels of triglycerides (TG, mmol/L), total cholesterol (TC, mmol/L), glycated hemoglobin (HbA1c), albumin (g/dl), high-density lipoprotein cholesterol (HDL, mmol/L), and low-density lipoprotein cholesterol (LDL, mmol/L).

### Outcome variables

Both gallstone incidence and age at first gallbladder surgery were evaluated with a questionnaire. In total, 1,381 participants provided information regarding their age at first gallbladder surgery, including 379 patients who underwent gallbladder surgery for reasons other than gallstones.

### Statistical analyses

NHANES sample weights were applied to account for the complex multi-stage cluster design of the underlying data. Categorical data were given as numbers (weighted percentages) and compared using chi-squared tests, whereas continuous variables were reported as means ± standard error (SE) and compared with Student’s *t*-tests. Odds ratios (ORs) and corresponding 95% confidence intervals (CIs) were calculated for the associations between individual dietary indices and gallstone incidence through univariate and multivariate-adjusted logistic regression approaches. These indices were treated as both continuous variables and categorical variables when conducting these weighted logistic regression analyses, separating values into quartiles in the latter case. Model 1 was unadjusted, while Model 2 was adjusted for age, sex, and ethnicity, and Model 3 was further adjusted for BMI, smoking history, physical activity, TG, TC, hyperlipidemia, hypertension, diabetes mellitus, contraceptives, estrogens, and fibrates. Potential nonlinear associations between these dietary indices and the prevalence of gallstones were tested through the use of restricted cubic spline (RCS) models. RCS curves were also used to examine the potential for nonlinearity with respect to associations between dietary indices and age at first gallbladder surgery. R (v 4.2.2) was used to conduct all statistical analyses, with *p* < 0.05 serving as the threshold to define significance.

## Results

### Study participants and baseline characteristics

In total, 12,426 adults in the USA were included in this analysis, of whom 1,370 had gallstones (Table 1). Average DII scores among patients with gallstones were higher than in healthy subjects (1.80 [0.06] vs. 1.42 [0.05]), whereas CDAI scores were lower among individuals with gallstones (0.37 [0.13] vs. 0.81 [0.08]). Individuals with gallstones were more likely to be older, female, smokers, individuals with higher BMI values, individuals engaged in less physical activity, and individuals with hyperlipidemia, hypertension, and diabetes mellitus. Participant characteristics in each DII and CDAI quartile are presented in Supplementary Tables S1, S2.

**Table 1:** Participant characteristics.

	Non-gallstones	Gallstones	*p* value
DII	1.42 (0.05)	1.80 (0.06)	<0.0001
CDAI	0.81 (0.08)	0.37 (0.13)	0.002
Age (years)	47.25 (0.43)	57.52 (0.50)	<0.0001
PIR	3.12 (0.04)	2.90 (0.06)	<0.001
Energy intake (kcal)	2175.65 (12.70)	1943.93 (29.65)	<0.0001
HbA1c	5.65 (0.01)	5.93 (0.03)	<0.0001
albumin (g/L)	41.17 (0.08)	39.75 (0.16)	<0.0001
TG (mmol/L)	1.26 (0.02)	1.41 (0.05)	0.003
TC (mmol/L)	4.89 (0.03)	4.88 (0.05)	0.89
HDL (mmol/L)	1.39 (0.01)	1.36 (0.01)	0.01
LDL (mmol/L)	2.85 (0.02)	2.87 (0.06)	0.73
BMI (kg/m^2^)	29.35 (0.15)	33.47 (0.39)	<0.0001
<25	2,931 (27.49)	162 (11.99)	
25–30	3,610 (32.07)	362 (27.41)	
>30	4,409 (39.83)	808 (58.63)	
Missing	106 (0.61)	38 (1.97)	
Sex (%)			<0.0001
Female	5,414 (49.36)	969 (72.75)	
Male	5,642 (50.64)	401 (27.25)	
Race (%)			0.02
Mexican American	1,341 (8.77)	167 (7.41)	
Non-Hispanic Black	2,925 (12.08)	270 (7.74)	
Non-Hispanic White	3,841 (61.59)	595 (66.38)	
Other Hispanic	1,062 (7.31)	154 (7.64)	
Other Race	1,887 (10.25)	184 (10.83)	
Educational status (%)			0.09
Less than high school	808 (3.45)	87 (2.92)	
High school	3,852 (34.55)	499 (38.36)	
More than high school	6,379 (61.94)	784 (58.72)	
Missing	17 (0.06)	0 (0.00)	
Physical activity (%)			<0.0001
No	5,722 (44.08)	840 (54.59)	
Moderate	2,550 (25.48)	326 (28.61)	
Vigorous	817 (8.70)	49 (3.23)	
Both	1,967 (21.74)	155 (13.56)	
Smoke (%)			<0.001
No	6,414 (58.41)	731 (52.35)	
Yes	4,640 (41.58)	637 (47.61)	
Missing	2 (0.01)	2 (0.04)	
Diabetes mellitus (%)			<0.0001
No	8,814 (84.90)	895 (72.89)	
Yes	2,125 (14.04)	467 (26.80)	
Missing	117 (1.06)	8 (0.30)	
Hypertension (%)			<0.0001
No	6,641 (65.48)	580 (45.17)	
Yes	4,406 (34.45)	789 (54.80)	
Missing	9 (0.07)	1 (0.03)	
Hyperlipidemia (%)			<0.0001
No	3,778 (35.30)	327 (23.68)	
Yes	7,275 (64.70)	1,043 (76.32)	
Missing	3 (0.00)	0 (0.00)	
Contraceptives (%)			0.67
No	10,776 (96.67)	1,343 (97.00)	
Yes	264 (3.20)	27 (3.00)	
Missing	16 (0.13)	0 (0.00)	
Estrogens (%)			0.67
No	10,749 (96.07)	1,327 (95.85)	
Yes	291 (3.80)	43 (4.15)	
Missing	16 (0.13)	0 (0.00)	
Fibrates (%)			0.36
No	10,946 (98.94)	1,355 (99.35)	
Yes	94 (0.93)	15 (0.65)	
Missing	16 (0.13)	0 (0.00)	

DII, dietary inflammatory index; CDAI, composite dietary antioxidant index; PIR, family poverty to income ratio; BMI, body mass index. [Categorical data were showed as the number of cases (weighted percentages), continuous variables were showed as means (standard error)].

### Associations between DII/CDAI and gallstone prevalent

To better understand how DII scores related to the incidence of gallstones, three multivariate logistic models were established (Table 2). DII values were significantly associated with gallstone prevalence under Model 1 (OR = 1.11; 95% CI, 1.07–1.16; *p* < 0.0001) and Model 2 (OR = 1.09; 95% CI, 1.04–1.13; *p* < 0.001). Under Model 3, the OR for this relationship was 1.10 (95% CI, 1.01–1.19) such that every unit increase in DII was associated with a 10% rise in the risk of gallstones. When DII was analyzed in quartiles, individuals in the highest quartile (Q4, corresponding to a diet with greater inflammatory potential) exhibited a significant increase in gallstone risk relative to individuals in Q1, although DII scores and gallstone rates were not related in Q2 or Q3 participants. The same association was noted under both Model 2 (adjusted for age, sex, ethnicity) and Model 3 (additionally adjusted for BMI, smoking status, TC, TG, physical activity, hyperlipidemia, hypertension, diabetes mellitus, contraceptives, estrogens, and fibrates).

**Table 2:** Logistic regression analyses of the link between dietary index values and the incidence of gallstones.

	Model 1		Model 2		Model 3	
	OR (95% CI)	*p* value	OR (95% CI)	*p* value	OR (95% CI)	*p* value
DII	1.11 (1.07, 1.16)	<0.0001	1.09 (1.04, 1.13)	<0.001	1.10 (1.01, 1.19)	0.03
Q1	Ref		Ref		Ref	
Q2	1.08 (0.88, 1.31)	0.47	1.05 (0.85, 1.29)	0.67	1.27 (0.85, 1.90)	0.22
Q3	1.33 (1.08, 1.63)	0.01	1.18 (0.97, 1.44)	0.09	1.32 (0.94, 1.84)	0.10
Q4	1.66 (1.42, 1.94)	<0.0001	1.52 (1.27, 1.82)	<0.0001	1.61 (1.08, 2.39)	0.02
*p* for tend		<0.0001		<0.001		0.02
CDAI	0.97 (0.95, 0.99)	0.004	0.98 (0.96, 0.99)	0.005	0.94 (0.91, 0.97)	0.001
Q1	Ref		Ref		Ref	
Q2	0.86 (0.69, 1.07)	0.17	0.86 (0.67, 1.10)	0.23	0.87 (0.62, 1.21)	0.38
Q3	0.72 (0.52, 1.01)	0.06	0.74 (0.51, 1.06)	0.10	0.99 (0.62, 1.58)	0.97
Q4	0.77 (0.64, 0.93)	0.01	0.79 (0.64, 0.97)	0.03	0.66 (0.47, 0.91)	0.02
*p* for tend		0.01		0.03		0.04

Model 1 had no covariate-adjusted.

Model 2 adjusted age, gender, and race.

Model 3 adjusted age, gender, race, physical activity, BMI, smoke, diabetes mellitus, hypertension, hyperlipidemia, TG, TC, contraceptives, estrogens, and fibrates.

This same multivariate approach was also employed to examine the association between CDAI values and the incidence of gallstones. Under Model 1, higher CDAI scores were related to a reduction in gallstone risk. Under Model 2 (adjusted for age, sex, ethnicity) and Model 3 (additionally adjusted for BMI, smoking status, TC, TG, physicalactivity, hyperlipidemia, hypertension, diabetes mellitus, contraceptives, estrogens, and fibrates) this same trend was also observed (Model 2: OR = 0.98, 95% CI, 0.96–0.99, *p* = 0.005; Model 3: OR = 0.94, 95% CI, 0.91–0.97, *p* = 0.001). When assessing CDAI scores in quartiles, individuals in CDAI Q4 exhibited a 34% (OR = 0.66; 95% CI, 0.47–0.91; *p* = 0.02) reduction in gallstone risk relative to those in Q1 under Model 3, whereas gallstone risk and CDAI scores were not significantly related in Q2 or Q3 relative to Q1.

**Figure 2:**
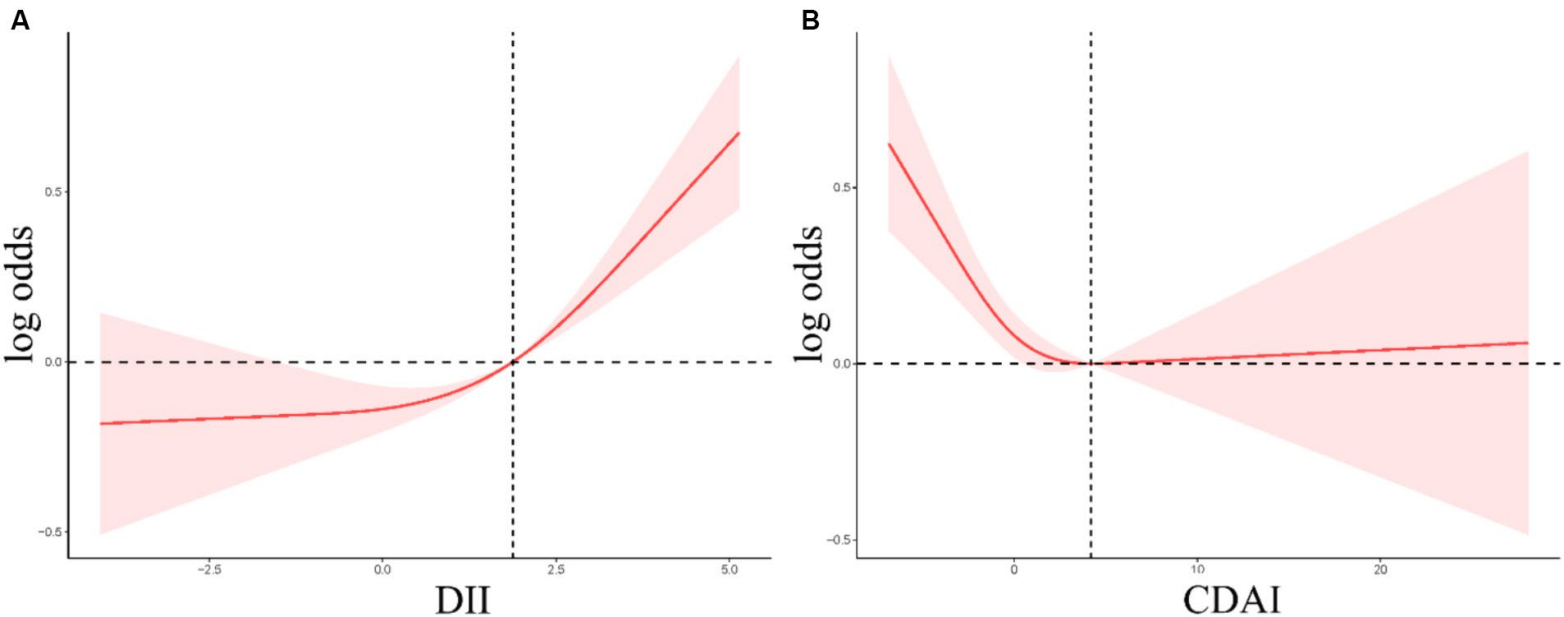
**(A,B)** RCS curves corresponding to the association between the incidence of gallstones and DII **(A)** or CDAI **(B)** values.

Smooth curve fitting revealed a positive non-linear relationship between DII values and gallstone incidence (Figure 2A), while for CDAI, a negative linear relationship with gallstone incidence was noted at CDAI values below 4.20, whereas gallstone risk remained stable when CDAI scores exceeded this threshold (Figure 2B).

Subgroup analysis was conducted to evaluate the consistency of the association between dietary indices and gallstones in different populations (Table 3). DII exhibited a positive correlation with gallstones in the majority of subgroups, whereas a significant association between CDAI and gallstones was solely observed in the Mexican American population and those with a less than high school educational status, no diabetes mellitus, no hypertension, and no use of contraceptives and fibrates. Moreover, The interaction between estrogens and the relationship between CDAI score and gallstone was significant (*p* for interaction = 0.03). Subgroup analyses did not detect any significant interactions between DII scores and gallstone incidence among the analyzed subgroups (*p* for interaction >0.05).

**Table 3:** Subgroup analyses focused on the relationship between dietary index values and the incidence of gallstones.

	DII			CDAI		
	OR (95% CI)	*p* value	*p* for interaction	OR (95% CI)	*p* value	*p* for interaction
BMI (kg/m^2^)			0.82			0.58
<25	1.13 (0.98, 1.30)	0.09		0.96 (0.89, 1.03)	0.20	
25–30	1.07 (1.00, 1.15)	0.04		0.99 (0.96, 1.03)	0.71	
>30	1.09 (1.01, 1.17)	0.02		0.98 (0.95, 1.01)	0.17	
Sex			0.24			0.62
Female	1.05 (1.00, 1.09)	0.03		0.98 (0.95, 1.00)	0.07	
Male	1.13 (1.00, 1.26)	0.04		0.96 (0.91, 1.02)	0.16	
Race			0.11			0.11
Non-Hispanic Black	1.14 (1.06, 1.22)	<0.001		1.00 (0.97, 1.03)	0.87	
Other Race	1.14 (0.91, 1.43)	0.25		0.99 (0.92, 1.07)	0.87	
Non-Hispanic White	1.08 (1.02, 1.14)	0.01		0.98 (0.95, 1.01)	0.21	
Mexican American	1.39 (1.27, 1.51)	<0.0001		0.89 (0.85, 0.93)	<0.0001	
Other Hispanic	1.23 (1.04, 1.45)	0.02		0.91 (0.82, 1.00)	0.05	
Educational status			0.42			0.33
Less than high school	1.31 (1.09, 1.57)	0.004		0.87 (0.79, 0.96)	0.01	
High school	1.10 (1.00, 1.21)	0.05		0.97 (0.93, 1.02)	0.26	
More than high school	1.11 (1.05, 1.17)	<0.001		0.98 (0.95, 1.01)	0.16	
Physical activity (%)			0.17			0.4
No	1.10 (1.02, 1.18)	0.02		0.97 (0.95, 1.00)	0.05	
Moderate	1.03 (0.94, 1.14)	0.51		1.00 (0.96, 1.04)	0.94	
Vigorous	0.92 (0.77, 1.11)	0.38		1.04 (0.96, 1.12)	0.32	
Both	1.19 (1.05, 1.36)	0.01		0.95 (0.88, 1.03)	0.24	
Smoke			0.98			0.72
No	1.11 (1.02, 1.20)	0.01		0.97 (0.94, 1.00)	0.07	
Yes	1.11 (1.04, 1.18)	0.001		0.98 (0.95, 1.01)	0.20	
Diabetes mellitus			0.67			0.82
No	1.10 (1.05, 1.15)	<0.0001		0.98 (0.96, 1.00)	0.03	
Yes	1.12 (1.03, 1.21)	0.01		0.97 (0.94, 1.01)	0.11	
Hypertension			0.88			0.03
No	1.11 (1.05, 1.17)	<0.001		0.95 (0.93, 0.98)	0.002	
Yes	1.10 (1.04, 1.17)	0.002		1.00 (0.97, 1.02)	0.68	
Hyperlipidemia			1			0.72
No	1.11 (1.02, 1.19)	0.01		0.97 (0.93, 1.01)	0.12	
Yes	1.11 (1.04, 1.17)	0.001		0.98 (0.95, 1.00)	0.09	
Contraceptives			0.05			0.09
No	1.11 (1.06, 1.15)	<0.0001		0.98 (0.96, 1.00)	0.02	
Yes	1.46 (1.09, 1.95)	0.01		0.82 (0.66, 1.02)	0.07	
Estrogens			0.23			0.03
No	1.11 (1.06, 1.15)	<0.0001		0.98 (0.96, 1.00)	0.03	
Yes	1.35 (0.96, 1.90)	0.08		0.82 (0.70, 0.97)	0.02	
Fibrates			0.06			0.63
No	1.11 (1.07, 1.15)	<0.0001		0.97 (0.95, 0.99)	0.005	
Yes	1.38 (1.08, 1.76)	0.01		0.93 (0.74, 1.16)	0.48	

### Higher DII scores are related to the need for gallbladder surgery at a younger age

**Figure 3:**
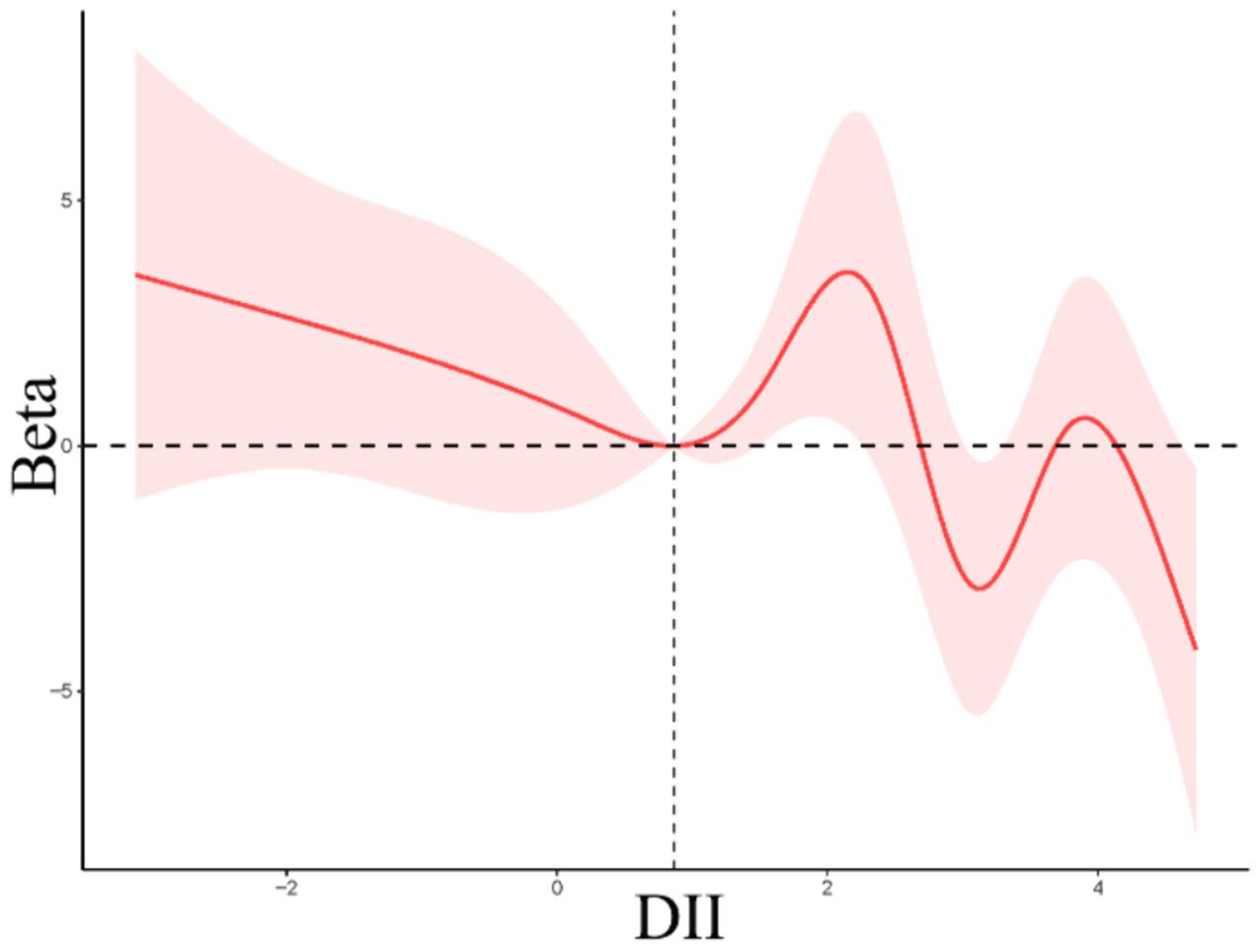
Restricted cubic spline curves for the relationships between DII values and age at first gallbladder surgery.

Linear regression analyses were next employed to examine the link between dietary indices and age at first gallbladder surgery in the NHANES cohort. A negative correlation was noted between DII scores and age at first gallbladder surgery (Table 4), with patients requiring surgery at a younger age, on average, with rising DII values. Smooth curve fitting indicated that this association was nonlinear, with a decrease in age at first surgery with increasing DII values at scores below 0.86, while above this breakpoint the curve was M-shaped with an overall downward trend (Figure 3). CDAI values were not significantly correlated with age at first gallbladder surgery.

**Table 4:** Linear regression analyses of the associations between dietary indices and age at first gallbladder surgery.

	*β*	Standard error	*p* value	95%CI
DII	−0.64	0.31	0.04	(−1.26, −0.02)
CDAI	0.2	0.13	0.13	(−0.06, 0.46)

DII, dietary inflammatory index; CDAI, composite dietary antioxidant index.

## Discussion

Here, the associations between dietary indices and gallstone incidence were assessed in a large representative cohort of adults in the USA. This analytical approach revealed an increase in gallstone risk among individuals with higher DII scores, whereas gallstone risk rose with lower CDAI scores even following adjustment for a range of covariates. Higher DII scores were also related to a younger age at first gallbladder surgery, whereas CDAI scores were unrelated to this outcome variable.

In a prior cross-sectional analysis of individuals in Iran (20), higher DII scores were related to a reduction in gallstone risk in direct contrast with the present results in the USA. DII values are computed based on the inflammatory potential of a given diet and these values have been linked to a range of inflammatory diseases (23, 24) including rheumatoid arthritis (25), nonalcoholic fatty liver (26–28), and *Helicobacter pylori* infections (29). Higher DII scores are also associated with the production of higher concentrations of TNF-α, IL-6, CRP, and a range of other factors that contribute to greater gallstone risk (30–32). A study of subjects in China reported an association between levels of IL-6 and IL-10 and the risk of gallstone development (7). Given that IL-10 and IL-6 are inflammatory biomarkers related to DII calculations (13), this supports the existence of a direct association between DII values and gallstone development. A prospective cohort analysis further demonstrated that CRP levels are independently associated with gallstone risk (33). The present data align well with past reports of strong positive relationships between DII, which serves as a comprehensive index for dietary inflammation, and gallstone incidence. Higher DII values are also related to the incidence of metabolic syndrome (34), obesity (35), and insulin resistance (36), all of which have the potential to directly or indirectly influence gallstone incidence (1, 34, 37), consistent with the positive relationship noted between DII and gallstones.

Higher CDAI scores, which correspond to a diet that is rich in antioxidants, were herein found to be related to a reduction in gallstone risk. Oxidative stress is firmly established as a regulator of gallstone development (18, 38, 39). For example, the intake of low levels of vitamin C (40), which is among the best-characterized antioxidant micronutrients, can contribute to a greater risk of gallstone formation as a result of the disruption of the production of free radicals involved in gallstone formation through changes in the protein and lipid content of the bile. Epithelial cells within the gallbladder secrete mucin, which is a glycoprotein. The secretion of overly high mucin levels in response to oxygen radicals has been reported to contribute to cholesterol destabilization and the biogenesis of gallstones (41). Antioxidant-rich diets can help abrogate gallstone risk, as supported by the present results (42, 43), which revealed a consistent negative association between CDAI values and the prevalence of gallstones in this study population (19). However, the stability of this correlation across diverse populations was not supported by the findings of the stratiffied analysis. The limited sample size in this study might have contributed to the absence of a significant negative association in the subgroup analysis.

The relationship between DII, CDAI, and gallstones indicated that a healthy dietary pattern characterized by low DII and high CDAI value has a protective effect on gallstones. This healthy dietary pattern consists of high consumption of vegetables, fruits, fiber-rich foods, and nuts and low consumption of high-calorie diet, polyunsaturated and monounsaturated fats (44–46). The correlation between the healthy dietary pattern and gallstones may be attributed to the biliary microbiota (10). The relationship between these dietary indices and age at first gallbladder surgery was additionally examined in this analysis. Higher DII values were related to the need for gallbladder surgery at a younger age. Specifically, for every increase in DII by one unit, the age of first gallbladder surgery was reduced by 0.64 years. This is the first article to report this relationship. While additional validation will be essential, this may suggest that efforts to modulate dietary composition at a younger age can better guard against gallstones and related adverse outcomes.

Limitations to this study include that this was a cross-sectional analysis such that the causal nature of associations between gallstone incidence and CDAI or DII values could not be established. Moreover, survey data were derived from questionnaires such that they may be limited by recall bias. In addition, the association between dietary indices and gallstone incidence could be influenced by other unconsidered or residual confounding factors. Lastly, limited sample sizes and poor statistical power may have contributed to some degree of uncertainty in particular subgroup analyses.

## Conclusion

In summary, the relationships between the CDAI and DII indices and the incidence of gallstones were evaluated for the general US population. These results suggest that gallstone risk increases when subjects consume a diet that is proinflammatory, as evidenced by a higher DII score, whereas antioxidant-rich diets with higher CDAI scores are conversely associated with a reduction in gallstone risk. This study is also the first to document an association between higher DII scores and the need to undergo gallbladder surgery at a younger age.

## Data availability statement

Publicly available datasets were analyzed in this study. This data can be found at: https://www.cdc.gov/nchs/nhanes.

## Ethics statement

The studies involving humans were approved by The National Center for Health Statistics Institutional Review Board. The studies were conducted in accordance with the local legislation and institutional requirements. The participants provided their written informed consent to participate in this study.

## Author contributions

CJ: Conceptualization, Data curation, Formal analysis, Funding acquisition, Investigation, Methodology, Project administration, Resources, Software, Supervision, Validation, Visualization, Writing – original draft, Writing – review & editing. YS: Conceptualization, Data curation, Formal analysis, Funding acquisition, Investigation, Methodology, Project administration, Resources, Software, Supervision, Validation, Visualization, Writing – original draft, Writing – review & editing.
