# Supplementary material for: Characterizing the relationships between dietary indices, gallstone prevalence and the need for gallbladder surgery in the general US population
Source: Front Nutr. 2024 May 7;11:1392960. doi: 10.3389/fnut.2024.1392960 (PMC11110571; doi:10.3389/fnut.2024.1392960)
Supplement: Supplementary file 1 [file Table_1.docx]

Supplementary Table S1：Participant characteristics according to the quartiles of DII.

|  | DII | | | |  |
| --- | --- | --- | --- | --- | --- |
|  | Q1 | Q2 | Q3 | Q4 | *p* value |
| Age (years) | 49.37 (0.53) | 47.60 (0.55) | 48.78 (0.57) | 47.24 (0.68) | 0.01 |
| PIR | 3.35 (0.05) | 3.10 (0.06) | 3.04 (0.07) | 2.69 (0.06) | < 0.0001 |
| Energyintake (kcal) | 2727.24 (28.47) | 2238.72 (26.47) | 1833.79 (28.27) | 1361.45 (21.35) | < 0.0001 |
| HbA1c | 5.65 (0.02) | 5.66 (0.03) | 5.72 (0.03) | 5.70 (0.02) | 0.19 |
| Albumin (g/L) | 41.50 (0.08) | 40.91 (0.08) | 40.85 (0.15) | 40.47 (0.18) | < 0.0001 |
| TG (mmol/L) | 1.31 (0.04) | 1.24 (0.03) | 1.37 (0.06) | 1.18 (0.03) | 0.03 |
| TC (mmol/L) | 4.82 (0.03) | 4.97 (0.04) | 4.93 (0.05) | 4.85 (0.05) | < 0.001 |
| HDL (mmol/L) | 1.40 (0.01) | 1.40 (0.02) | 1.38 (0.01) | 1.35 (0.01) | 0.05 |
| LDL (mmol/L) | 2.77 (0.03) | 2.94 (0.05) | 2.89 (0.05) | 2.84 (0.05) | 0.01 |
| BMI (kg.m2) | 28.91 (0.20) | 30.05 (0.26) | 30.31 (0.24) | 30.45 (0.32) | < 0.0001 |
| <25 | 1062 (28.49) | 759 (24.46) | 655 (23.56) | 617 (25.24) |  |
| 25-30 | 1392 (35.12) | 1008 (30.74) | 856 (29.72) | 716 (28.41) |  |
| >30 | 1345 (35.65) | 1393 (44.22) | 1260 (45.82) | 1219 (45.45) |  |
| Missing | 41 (0.73) | 31 (0.58) | 37 (0.90) | 35 (0.90) |  |
| Sex (%) |  |  |  |  | < 0.0001 |
| Female | 1560 (40.99) | 1575 (51.82) | 1595 (58.45) | 1653 (64.27) |  |
| Male | 2280 (59.01) | 1616 (48.18) | 1213 (41.55) | 934 (35.73) |  |
| Race (%) |  |  |  |  | < 0.0001 |
| Mexican American | 570 (9.87) | 403 (8.88) | 304 (8.21) | 231 (6.52) |  |
| Non-Hispanic Black | 763 (8.37) | 799 (11.46) | 802 (13.16) | 831 (15.73) |  |
| Non-Hispanic White | 1341 (61.97) | 1172 (63.39) | 990 (61.90) | 933 (60.92) |  |
| Other Hispanic | 387 (7.87) | 304 (6.97) | 283 (6.63) | 242 (7.75) |  |
| Other Race | 779 (11.92) | 513 (9.30) | 429 (10.10) | 350 (9.07) |  |
| Educational status (%) |  |  |  |  | < 0.0001 |
| Less than high school | 282 (3.29) | 203 (2.94) | 219 (3.51) | 191 (4.04) |  |
| High school | 1076 (25.92) | 1058 (35.16) | 1078 (39.68) | 1139 (45.49) |  |
| More than high school | 2475 (70.71) | 1926 (61.88) | 1511 (56.81) | 1251 (50.37) |  |
| Missing | 7 (0.09) | 4 (0.01) | 0 (0.00) | 6 (0.10) |  |
| Physical activity (%) |  |  |  |  | < 0.0001 |
| No | 1671 (35.06) | 1628 (44.47) | 1603 (51.30) | 1660 (57.57) |  |
| Moderate | 948 (27.14) | 768 (26.80) | 661 (26.71) | 499 (21.15) |  |
| Vigorous | 298 (9.21) | 240 (8.48) | 182 (7.14) | 146 (6.67) |  |
| Both | 923 (28.59) | 555 (20.25) | 362 (14.85) | 282 (14.62) |  |
| Smoke (%) |  |  |  |  | < 0.0001 |
| No | 2274 (59.21) | 1884 (62.30) | 1563 (53.99) | 1424 (53.15) |  |
| Yes | 1565 (40.78) | 1307 (37.70) | 1243 (45.96) | 1162 (46.85) |  |
| Missing | 1 (0.01) | 0 (0.00) | 2 (0.05) | 1 (0.01) |  |
| Diabetes mellitus (%) |  |  |  |  | 0.01 |
| No | 3080 (85.34) | 2505 (84.17) | 2130 (81.54) | 1994 (81.89) |  |
| Yes | 714 (13.69) | 655 (14.64) | 652 (17.33) | 571 (17.59) |  |
| Missing | 46 (0.97) | 31 (1.19) | 26 (1.13) | 22 (0.53) |  |
| Hypertension (%) |  |  |  |  | 0.05 |
| No | 2339 (64.50) | 1883 (64.46) | 1550 (59.67) | 1449 (63.35) |  |
| Yes | 1500 (35.50) | 1306 (35.50) | 1255 (40.25) | 1134 (36.46) |  |
| Missing | 1 (0.00) | 2 (0.04) | 3 (0.08) | 4 (0.19) |  |
| Hyperlipidemia (%) |  |  |  |  | 0.06 |
| No | 1336 (36.95) | 1054 (34.46) | 936 (32.22) | 779 (30.20) |  |
| Yes | 2502 (63.04) | 2136 (65.54) | 1872 (67.78) | 1808 (69.80) |  |
| Missing | 2 (0.01) | 1 (0.00) | 0 (0.00) | 0 (0.00) |  |
| Contraceptives (%) |  |  |  |  | 0.002 |
| No | 3763 (97.54) | 3083 (95.14) | 2740 (97.19) | 2533 (96.81) |  |
| Yes | 71 (2.29) | 105 (4.78) | 63 (2.72) | 52 (3.10) |  |
| Missing | 6(0.17) | 3 (0.08) | 5 (0.09) | 2 (0.09) |  |
| Estrogens (%) |  |  |  |  | 0.001 |
| No | 3740 (96.79) | 3079 (94.46) | 2722 (95.76) | 2535 (97.23) |  |
| Yes | 94 (3.04) | 109 (5.46) | 81 (4.15) | 50 (2.69) |  |
| Missing | 6 (0.17) | 3 (0.08) | 5 (0.09) | 2 (0.09) |  |
| Fibrates (%) |  |  |  |  | 0.17 |
| No | 3799 (98.70) | 3148 (98.76) | 2782 (99.29) | 2572 (99.48) |  |
| Yes | 35 (1.13) | 40 (1.16) | 21 (0.62) | 13 (0.44) |  |
| Missing | 6 (0.17) | 3 (0.08) | 5 (0.09) | 2 (0.09) |  |
