# Supplementary material for: Characterizing the relationships between dietary indices, gallstone prevalence and the need for gallbladder surgery in the general US population
Source: Front Nutr. 2024 May 7;11:1392960. doi: 10.3389/fnut.2024.1392960 (PMC11110571; doi:10.3389/fnut.2024.1392960)
Supplement: Supplementary file 2 [file Table_2.docx]

Supplementary Table S2：Participant characteristics according to the quartiles of CDAI.

|  | CDAI | | | |  |
| --- | --- | --- | --- | --- | --- |
|  | Q1 | Q2 | Q3 | Q4 | *p* value |
| Age (years) | 48.07 (0.71) | 48.81 (0.53) | 48.68 (0.51) | 48.04 (0.73) | 0.56 |
| PIR | 2.71 (0.07) | 2.95 (0.06) | 3.23 (0.05) | 3.29 (0.07) | < 0.0001 |
| Energyintake (kcal) | 1255.79 (16.03) | 1821.37 (15.52) | 2205.85 (21.49) | 2819.50 (26.41) | < 0.0001 |
| HbA1c | 5.72 (0.03) | 5.72 (0.02) | 5.66 (0.02) | 5.65 (0.03) | 0.37 |
| albumin(g/L) | 40.72 (0.18) | 40.96 (0.14) | 41.19 (0.10) | 41.06 (0.08) | 0.05 |
| TG (mmol/L) | 1.31 (0.05) | 1.26 (0.03) | 1.26 (0.04) | 1.29 (0.04) | 0.72 |
| TC (mmol/L) | 4.96 (0.05) | 4.88 (0.04) | 4.88 (0.04) | 4.86 (0.03) | 0.19 |
| HDL (mmol/L) | 1.38 (0.01) | 1.38 (0.02) | 1.39 (0.01) | 1.40 (0.01) | 0.32 |
| LDL (mmol/L) | 2.94 (0.05) | 2.82 (0.03) | 2.85 (0.03) | 2.83 (0.03) | 0.12 |
| BMI (kg/m2) | 30.38 (0.23) | 29.85 (0.32) | 29.70 (0.16) | 29.51 (0.26) | 0.05 |
| <25 | 663 (24.72) | 656 (24.63) | 780 (25.20) | 994 (27.59) |  |
| 25-30 | 795 (29.06) | 881 (30.51) | 1063 (32.90) | 1233 (32.52) |  |
| >30 | 1179 (45.43) | 1222 (43.77) | 1353 (41.28) | 1463 (39.25) |  |
| Missing | 41 (0.79) | 29 (1.09) | 39 (0.62) | 35 (0.64) |  |
| Sex (%) |  |  |  |  | 0.07 |
| Female | 1474 (56.39) | 1397 (50.38) | 1585 (50.00) | 1927 (52.09) |  |
| Male | 1204 (43.61) | 1391 (49.62) | 1650 (50.00) | 1798 (47.91) |  |
| Race (%) |  |  |  |  | < 0.0001 |
| Mexican American | 251 (6.88) | 346 (9.53) | 394 (7.99) | 517 (9.55) |  |
| Non-Hispanic Black | 868 (16.32) | 745 (12.93) | 714 (9.17) | 868 (10.09) |  |
| Non-Hispanic White | 861 (58.23) | 1015 (61.61) | 1241 (64.63) | 1319 (62.57) |  |
| Other Hispanic | 290 (8.65) | 245 (7.00) | 314 (6.17) | 367 (7.82) |  |
| Other Race | 408 (9.93) | 437 (8.93) | 572 (12.04) | 654 (9.98) |  |
| Educational status (%) |  |  |  |  | < 0.0001 |
| Less than high school | 254 (4.87) | 210 (3.91) | 211 (2.85) | 220 (2.67) |  |
| High school | 1181 (45.62) | 1043 (38.91) | 1010 (31.97) | 1117 (28.94) |  |
| More than high school | 1237 (49.40) | 1533 (57.17) | 2010 (65.16) | 2383 (68.32) |  |
| Missing | 6 (0.11) | 2 (0.01) | 4 (0.02) | 5 (0.07) |  |
| Physical activity (%) |  |  |  |  | < 0.0001 |
| No | 1657 (54.75) | 1568 (51.28) | 1626 (42.81) | 1711 (38.04) |  |
| Moderate | 531 (21.34) | 656 (23.60) | 823 (29.84) | 866 (26.44) |  |
| Vigorous | 180 (7.97) | 185 (8.23) | 222 (7.56) | 279 (8.53) |  |
| Both | 310 (15.94) | 379 (16.89) | 564 (19.79) | 869 (27.00) |  |
| Smoke (%) |  |  |  |  | < 0.0001 |
| No | 1412 (48.68) | 1613 (59.11) | 1902 (61.21) | 2218 (59.06) |  |
| Yes | 1265 (51.31) | 1173 (40.84) | 1333 (38.79) | 1506 (40.93) |  |
| Missing | 1 (0.01) | 2 (0.05) | 0 (0.00) | 1 (0.01) |  |
| Diabetes mellitus (%) |  |  |  |  | 0.02 |
| No | 2031 (81.11) | 2136 (82.31) | 2517 (83.47) | 3025 (85.85) |  |
| Yes | 628 (18.03) | 629 (17.09) | 685 (15.51) | 650 (12.91) |  |
| Missing | 19 (0.86) | 23 (0.61) | 33 (1.01) | 50 (1.25) |  |
| Hypertension (%) |  |  |  |  | 0.02 |
| No | 1477 (62.04) | 1536 (60.00) | 1915 (63.85) | 2293 (65.48) |  |
| Yes | 1198 (37.83) | 1248 (39.86) | 1318 (36.11) | 1431 (34.52) |  |
| Missing | 3 (0.13) | 4 (0.14) | 2 (0.04) | 1 (0.00) |  |
| Hyperlipidemia (%) |  |  |  |  | 0.15 |
| No | 813 (30.32) | 930 (34.06) | 1033 (35.14) | 1329 (35.11) |  |
| Yes | 1865 (69.68) | 1858 (65.94) | 2201 (64.86) | 2394 (64.88) |  |
| Missing | 0 (0.00) | 0 (0.00) | 1 (0.00) | 2 (0.01) |  |
| Contraceptives (%) |  |  |  |  | 0.09 |
| No | 2629 (97.90) | 2718 (95.84) | 3160 (97.24) | 3612 (96.15) |  |
| Yes | 45 (1.98) | 68 (4.10) | 71 (2.68) | 107 (3.68) |  |
| Missing | 4 (0.11) | 2 (0.06) | 4 (0.08) | 6 (0.17) |  |
| Estrogens (%) |  |  |  |  | 0.03 |
| No | 2628 (97.61) | 2706 (95.08) | 3139 (96.62) | 3603 (95.32) |  |
| Yes | 46 (2.27) | 80 (4.86) | 92 (3.30) | 116 (4.51) |  |
| Missing | 4 (0.11) | 2 (0.06) | 4 (0.08) | 6 (0.17) |  |
| Fibrates (%) |  |  |  |  | 0.39 |
| No | 2658 (99.35) | 2761 (99.25) | 3195 (98.92) | 3687 (98.68) |  |
| Yes | 16 (0.54) | 25 (0.69) | 36 (1.00) | 32 (1.15) |  |
| Missing | 4 (0.11) | 2 (0.06) | 4 (0.08) | 6 (0.17) |  |
